# Using the Five-Microskills Method in Veterinary Medicine Clinical Teaching

**DOI:** 10.3390/vetsci8060089

**Published:** 2021-05-24

**Authors:** Amanda Nichole (Mandi) Carr, Roy Neville Kirkwood, Kiro Risto Petrovski

**Affiliations:** 1 Davies Livestock Research Centre, School of Animal and Veterinary Sciences, The University of Adelaide, Roseworthy, SA 5371, Australia; mandi.carr@adelaide.edu.au; 2 Swine Production Medicine, School of Animal and Veterinary Sciences, The University of Adelaide, Roseworthy, SA 5371, Australia; roy.kirkwood@adelaide.edu.au; 3 Australian Centre for Antimicrobial Resistance Ecology, School of Animal and Veterinary Sciences, The University of Adelaide, Roseworthy, SA 5371, Australia

**Keywords:** deep learning, student-focused education, workplace training techniques, veterinary training tool

## Abstract

Effective clinical teaching is essential for the development of veterinary learners. Teaching clinical reasoning is a challenge for veterinary instructors as many lack adequate training in clinical teaching. In this paper, we propose the use of the five-microskills (FMS; also known as the one-minute preceptor) model of clinical teaching as a tool that can be used not only in teaching during clinical encounters but also during traditional teaching sessions (e.g., practicals). The FMS model assists the instructor in estimating the level of knowledge and development of the learner and allows for providing feedback. The FMS model is applicable in the busy clinical or teaching schedule of the instructor and requires training only of the instructor, not the learner. We provide two examples of the use of the FMS model, one of a clinical encounter and the other a biochemistry practical. From the examples, readers should be able to extract the basis of the model and start using it in their day-to-day practice. For proper use of the model, 1–4 h of training is usually recommended.

## 1. Introduction

Although a significant proportion of veterinary medical education occurs in clinical settings, the literature describing teaching strategies in this setting are limited. Therefore, we felt that this article would facilitate a discussion of research in this field. Clinical teaching is an essential part of the education of veterinary medicine learners, particularly for development and application of their ‘clinical reasoning’. Clinical reasoning is recognized as a critical skill to develop and a vital clinical competence [1,2,3]. To achieve success, clinical teaching requires commitment by all involved parties: the staff in the clinical environment, learners, instructors, and involved client/patient. Exposure to clinical practice allows learners to advance their leadership skills and professional standards, improve their communication skills, deepen their appreciation of practice management, and work within economic constraints [3,4]. Clinical teaching should be implemented in a ‘proper learning environment’ (real work context), allowing learners to be stimulated to participate in clinical discussions, develop confidence, and obtain competence in as many Day One skills as possible. The learning environment should also facilitate an interest into exploring topics out of the learner’s comfort zone and allow for peer-generated questioning and challenging [5,6]. Case management, led by the learner who takes case responsibility, often as a part of a team, must be under careful and extensive supervision and with a support network [3,4]. Many veterinary practitioners would enjoy being involved in, and are enthusiastic about, clinical teaching, a particularly fulfilling element being the joy of giving something back to the profession. However, the disruption that may result from clinical teaching [7] and the lack of understanding of the requirements of clinical teaching are important impediments to practitioner involvement. Veterinary medicine clinical instructors (instructors hereafter) should use any opportunity to expand the clinical reasoning of their learners. This may be achieved through providing them with new information (e.g., a mini lecture), although rote information is easily forgotten. An improved approach to clinical teaching is to expand the clinical reasoning of learners by facilitating a new way of thinking (stimulating what is recommended by the ‘deep learning’ theory) [8,9,10].

Currently, veterinary instructors rarely, if ever, receive official training in clinical teaching, and there are no standardized requirements. This has been identified as an important gap in both veterinary [1,11] and human medical education [11,12,13], including the learners’ opinion of stressors [14]. In the existing environment, each instructor utilizes their own way of clinical teaching [15]. Typically, instructors use their previous experience to structure their teaching, drawing on mentors and instructors they found to be useful during their own education and professional development. However, an instructor’s duties expand far beyond clinical knowledge to include assessment, guidance, role-modelling, support, and teaching [2]. Training of instructors by personnel with at least 2–3 years’ experience in using the model should encourage changes in teaching behaviors and effectiveness and better prepare instructors in teaching strategies (e.g., adult learning principles, teaching evidence-based content, and teaching in the presence of the client/patient) with the aim of improving their confidence to teach [2,3,13,16,17]. Particular topics to focus on include training in assessment, engagement and motivation of learners, management of conflicts, promotion of clinical reasoning, provision of feedback, and ways to minimize the disruption of the busy practice schedule [2,7,11,16].

Clinical teaching is based on principles of higher-order thinking skills such as analyzing, evaluating, and formulating, reaching beyond simply understanding and remembering. Using the principles of andragogy, learners are encouraged to develop autonomy and to become aware of and take responsibility for their own learning [1]. This is best achieved when working in teams; thus, clinical teaching in veterinary medicine should encourage team work [1]. The higher-order learning skills fall into three different categories: (1) higher-order learning skills in terms of transfer (learners acquire knowledge and are able to apply it in new situations); (2) higher-order learning skills in terms of critical thinking (learners make specific inquiries, explore viewpoints, and apply judgment); and (3) higher-order learning skills in terms of problem solving (a learner may not know the appropriate pathway to solve the enquiry but is capable of working to achieve the goal using general skills and avoids the need to memorize unnecessary steps) [18]. When teaching clinical or non-clinical skills, instructors need to be cognizant of modern student strengths and limitations. Generational changes require adjustment of clinical teaching to suit the learners’ requirements [19,20]. The learners of today have grown up in the internet age with most information available on-demand. Additionally, they prefer broadness rather than depth in their learning experience [19]. As such, modern learners are likely to have a relatively short attention span, so interactions will need to be concise [21,22]. Finally, the majority of modern learners are prone to multitasking, which might make it difficult for the clinical instructor from an older generation [20].

Implementation of strategic questioning in clinical teaching interactions stimulates clinical reasoning and other forms of higher thinking in learners [5,23], although causation could not be proven in the single study available in veterinary medicine [1]. Unfortunately, in that study, neither promotion of higher-level learning by instructors nor evidence of higher-level thinking by learners were detected, despite involving clinical rotations in the final year of the veterinary degree.

Strategic questioning usually originates from the instructors, but peer-generated questions should also be encouraged. Indeed, peer-generated questions reduce the pressure on the instructor to build-up a bank of suitable queries [24] and assist learners in developing independent higher-order thinking skills [24,25,26]. Therefore, when properly executed, strategic questioning is a strong facilitator of self-directed learning [10,26]. However, as self-directed learning may be more advantageous for learners at a more advanced level [10], it is assumed that peer-generated questioning is mainly applicable to advanced learners. In contrast, the current authors have used peer-generated questioning with learners in earlier levels, and anecdotal evidence suggests that it does work throughout the curriculum (e.g., clinician, learner, and teacher feedback and student assessment reviews). We also have implemented peer-creation of multiple-choice questions (MCQs) and used some of these questions during official exams. As with many other activities, learners engaged with this activity to different levels and experienced significant difficulties, similar to previous reports [27]. The quality of questions was not related to the final grade of the learner, but the engagement by learners with the course was better for those that were creating the MCQs throughout the year, compared with batch producers of questions, again similar to previous findings [25].

Scaffolding strategic questioning is essential in order to adjust educational experience to the skills and knowledge of learners. In a ‘proper learning environment’ enquiries just below and just above the level the of learner’s knowledge should be used occasionally. This stimulates learner involvement and deep-learning, although constant use of enquiries above the level of the learners’ knowledge was counter-productive, as learners lose motivation and become frustrated and distant from the course and the instructor [28].

Feedback is a purposeful conversation between the learner and instructor within a context and a culture with the aim of stimulating self-reflection, this being a powerful tool for deep learning. To be effective, feedback must be relevant, specific, timely, thorough, and offered in a constructive manner using descriptive rather than evaluative language with the aim of enhancing the learner’s performance. Without effective feedback, the progress of learners towards competence can be impeded [8,29,30]. However, a conversation including feedback may be challenging for both involved parties. Instructors may suspect that constructive criticism will influence their relationship with the learner or the learner’s self-esteem [8,29]. Indeed, many instructors have no formal training in the provision of feedback, so they may feel uncomfortable providing effective feedback [11,13,16,31,32,33].

Learners in veterinary medicine preferred honest constructive feedback, allowing for recognition of limitations to skills, knowledge, and behaviors [1]. Learners prefer instructors that provide them with effective feedback [30,34]. Feedback in clinical training of any medical profession, including veterinary medicine, is important for providing the learner with an opportunity to hear (read) about his/her performance, thus allowing them to gauge improvement over time. Only with effective feedback can a learner easily achieve Day One Competencies. Learners not provided with effective feedback would assume that they are doing well and have no areas for development and/or improvement [29,30].

Simply telling a learner that he/she did well/did not perform to their best is not sufficient. Effective feedback should be behavior-specific, selective, and timely [29]. The corrective feedback should provide the rationale for the recommended correction (e.g., how it has affected others). However, this does not mean that re-enforcing feedback should lack rationale.

Providing personalized feedback in front of a group could feel awkward. However, in clinical teaching in most cases, instructors should not be reluctant to provide the feedback to learners eager to receive constructive feedback, even in front of the group. Effective corrective feedback to one learner can provide a learning opportunity to all learners present [35]. Positive feedback should build self-esteem in the learner and stimulate the repetition and enhancement of the praised behavior(s) [2,7,29,36].

Case presentation is an important clinical teaching activity [37]. However, when presenting cases, students will tend to focus primarily on factual information, thus revealing little about their clinical reasoning or, indeed, their uncertainties [38]. There is a lack of standardized requirements for clinical teaching, so an aim for the instructor should be to employ a teaching model that prompts students to provide these details. A solution to the lack of standardized requirements for clinical teaching in veterinary medicine, and the involvement of novice instructors in that teaching, would be to use established medical teaching models that structure the learning experience of learners and the way instructors deliver the teaching. A few models are available in human medicine, such as concept mapping, the SNAPPS model (Summarize information; Narrow the differential; Analyze the differentials through compare and contrast; Probe the instructor to clarify uncertainty; Plan the management; Select a case for self-directed learning), and the five-microskills (FMS; also known as the One-Minute Preceptor) model. These, and other models, aim to improve clinical teaching efficiency and effectiveness through a structured and standardized learning experience [39]. Taking into consideration the OneHealth approach, models used in human medicine should be applicable in veterinary medicine. Medical education literature has a large body of evidence indicating that some of these models work in various fields of medicine, including nursing. The largest body of evidence exists for the utility of the FMS model [34,35,36,39]. Additionally, data regarding other aspects are available for veterinary medicine and agree with the human medicine findings (e.g., effective learning occurred in a positive learning environment and in situations where feedback was provided) [1]. Unfortunately, there is a dearth of evidence regarding the efficacy of any of the clinical teaching models in veterinary medicine.

Using information from various branches of human medicine, the FMS model was deemed to be an effective model for quality clinical teaching [34,35], resulting in favorable experiences for both learners and instructors [7]. Based on professional evaluations and learner feedback, instructors trained in the FMS model were, in general, ‘better educators’ compared with non-trained instructors or those using a ‘traditional clinical teaching model’ [40]. What learners really liked was the effective feedback and the assessment of clinical reasoning [34]. In a systematic review, it was reported that 5 of 12 studies showed significant improvements in the feedback given by instructors who were trained in the FMS model and in 4 of 12 studies, trained instructors were evidently better at assessing clinical reasoning of learners, particularly the differential diagnosis list, management plans, and presentation of disease scenarios [34]. Improvements have been reported for both novice and experienced instructors [2]. Interestingly, while learners liked the feedback, the model did not train the instructors in providing effective feedback. The preference of learners for a particular model was inconclusive, although it appeared that learners preferred the FMS model of clinical teaching over traditional approaches (memorization and recitation techniques) [34,40]. What learner’s preferred about the model were involvement in the decision-making process and the quality of feedback [40]. The FMS model was suitable for one-to-one and group clinical teaching [35]. What is important is that the use of the FMS model did not slow down clinical interaction which, overall, required less time than traditional clinical teaching [7]. Moreover, the FMS model has been utilized with success in the teaching of practical sessions in various medical disciplines [40,41]. Therefore, in continuation of this article, we will discuss only the FMS teaching model. We hope that training in this model will encourage a deeper-level of learning by learners, including earlier development of ‘illness scripts’, which is recognized as a deficiency in veterinary clinical teaching [1]. It is important to note that none of the models used in clinical teaching are yet to employ good methods for assessment of the competency domains (e.g., communication, empathy, ethics, and professionalism). It is not our intention to suggest the replacement of existing teaching skills but, rather, to describe alternative or additional tools that can be easily implemented in veterinary clinical and non-clinical teaching interactions.

## 2. The Five Microskills Model

The FMS model, also known as the one-minute preceptor (OMP) model, was developed by Neher et al. [36]. It provides a five-step (microskill) structure for clinical education: (1) Get a commitment; (2) Probe for supporting evidence; (3) Teach general rules; (4) Reinforce what was done well; and (5) Correct mistakes [36]. This model is not intended to be static but rather its’ versatility allows for the order of the microskills to be flexible and applied as required according to the specific needs of a particular clinical intervention [42,43]. Indeed, the versatility of the FMS model allows it to consider student learning preferences (visual, auditory, tactile) which, in turn, helps instructors move beyond their own comfort level in regard to teaching preference styles [44]. Visual learners can work through the sequence of events by creating a flow chart or decision tree. Auditory learners have the ability to discuss the events with their peers, and tactile learners have the option to move around during the teaching encounter or use a whiteboard to emphasize important relationships discovered within the encounter [14]. Table 1 indicates similar strategies that can facilitate different learner types [14].

In 1980, 18 major modes and microskills were identified by Koe and Vivian but using them in routine teaching was cumbersome. The FMS model extracts only 5 of the 18 major identified modes and microskills [43,45]. From an educationalist perspective, the FMS model incorporates features of Bruner’s theory of constructivism, Kolb’s theory of experiential learning, Knowles’ principles of teaching andragogy, and Schön’s theory of reflective practice [8,9,46,47,48]. The first two microskills are based on building on previous learning and the application of knowledge [9,46,47]. The third microskill is based on the principles of andragogy with presentation of general rules, ‘pearls of wisdom’, ‘rules of thumb’, or ‘take home points’ with an immediate relevance to the learner [2,46,49]. The last two skills are also based on the principles of andragogy related to the provision of feedback and stimulation of self-reflection and self-directed learning [2,8,9,46,48]. Bidirectional verbalization of the positive and corrective feedback in the last two skills is important for helping the learner to structure or re-structure previous and newly acquired knowledge [39,40]. Using these microskills, through a collaboration, should be educational for both the learner and the instructor [2,8,50]. Some authors state that the model is particularly suitable for novice instructors [43] while others were of the opinion that the model was suitable for all instructors regardless of their clinical teaching experience [34].

Using the FMS model effectively will provide the instructor with succinct guidelines of how to approach a clinical interaction, while the learner is assisted in the development of his/her clinical reasoning. The aim of the model is to facilitate development and usage of higher-thinking skills by the learner (e.g., clinical reasoning) and to stimulate their self-directed learning [2,36]. Importantly, as the FMS model is implemented at the time of the clinical interaction, it is verbal. Hence, it utilizes the ‘think out loud’ strategy known to stimulate self-assessment, self-reflection, and deeper learning [2,51]. One of the skills that learners recognize often as ‘not being their strong suit’ is communication [1]. The ‘think out loud’ strategy is therefore important for professional development of learners. For the instructor, the ‘think out loud’ strategy allows them to assess the learner’s knowledge and clinical reasoning [2]. The additional benefit for the learner involved with the FMS model is the ongoing feedback and access to immediate supervision [35].

A major advantage of the FMS model is the need for only a short training of instructors (1–4 h) compared with a few hours for both learners and instructors in SNAPPS, a few days in the concept mapping model, and 2 or 3 half days for the traditional model of clinical teaching [36,39,49]. The FMS model should be appropriately introduced to instructors (e.g., training provided), but there is no need for it to be introduced to learners [35]. This does not mean that learners should not receive specific training in clinical reasoning; indeed, the need for specific training of learners in clinical reasoning has been identified previously [1]. In contrast, the SNAPPS model, where the clinical teaching interaction is learner-driven, requires both instructor and learner to be trained to use the framework [35]. Interestingly, instructors prefer face-to-face training rather than an on-line self-study module, both in veterinary [16] and human medicine [13,52].

### 2.1. Get a Committment

This microskill should be used in a non-threatening way to get a commitment by the learner to approach the clinical encounter and arrive to a diagnosis or a plan: either further work up or a case management plan. Used early in the clinical interaction, the learner should collect data, start problem solving, and feel responsibility for the case and the need for collaboration. One to three questions should be enough to ‘get a commitment’. Asking too many questions would mean that the instructor has taken-over the case. This microskill should stimulate the learner to stray beyond their level of comfort. Thus, this microskill makes the clinical interaction more active and personal. Regular use of the ‘get a commitment’ microskill may increase the learner’s motivation for self-directed studies, as they will be aware it is an expectation to generate an independent blueprint for managing the presented case [49]. Specific questions to ask include:

- *‘How do you think we could manage this case?’*
- *‘What would you do if I were not here?’*
- *‘What do you think is going on here?’*
- *‘What further data collection would you do?’*

Used later in the clinical interaction with other microskills, it stimulates the learner’s self-directed learning and self-assessment. For example, when the learner has answered correctly or has shown correct behaviors, the ‘get a commitment’ microskill can be used to reinforce the positive behavior.

- *‘Based on the great health interview information you obtained, what parts of the clinical examination should we focus on?’*

However, when the response or behaviors are negative, the ‘get a commitment’ microskill can be used to encourage further work and an opportunity to correct the negative behaviors.

- *‘XXX. Now, when we agreed on the XX and with aim to prevent YY recurrence in the future clinical encounters, can you think of what you would do differently?’*

When a learner struggles with this microskill, it may be because he/she lacks didactic or content knowledge or experience in clinical reasoning, or he/she is afraid of the risk of being wrong and/or being evaluated. To put the learner at ease, the instructor may use a general statement such as *‘I am not asking you to get involved with this case to assess you.’* or *‘It is my intention to see your way of thinking and reasoning as that would help me become a better clinical instructor.’*

### 2.2. Probe for Supporting Evidence

This microskill should be used to test a learner’s knowledge and approach to the assessment and their arrival at a diagnosis or a management plan. It should assist the instructor in understanding the reasoning process of the learner and in detecting strengths and weaknesses. This microskill allows for assessment of the clinical reasoning of the learner. The microskill ‘probe for supporting evidence’ is essential to any clinical teaching interaction, as an appropriate diagnosis or management plan may be due to pure luck. Additionally, learners are prone to prematurely ‘jump to conclusions’ rather than working through the problem solving steps [1]. Indeed, a proportion of learners expressed their preference to be mentored on the proper clinical reasoning procedure [1]. By inclusion of this microskill, the instructor will find out where learners need mentoring.

- *‘Can you explain what elements of the health interview you considered in the selection of this diagnosis?’*
- *‘Can you explain why you would take this action first?’*
- *‘I would be very interested to hear your reasoning behind the choice of this medication?’*
- *‘I would like to hear what led you to that conclusion?’*
- *‘Please elaborate on why you feel it is important to focus on this part of the clinical examination.’*
- *‘What other differentials were considered for this case but were discarded, and why?’*

During discussion, a learner may often pause, expecting instructor assistance or seeking instructor approval/rejection. This is where the instructor should suppress the urge to ‘jump in with the explanation’ or ‘fill in the blank’. Only in this way does an instructor provide an opportunity for a learner to interpret the data.

Based on this microskill, the instructor should be able to vary instructions to be at the learner’s current learning level. When more than one learner is involved with a particular clinical interaction and they have different levels of understanding, the instructor should target most of the instructions at the lowest level of understanding in the group.

### 2.3. Teach General Rules

This microskill should be used as an opportunity for the instructor to briefly share his/her experience to make a significant impact on the learner. However, this should not become a lecture or session with information overload. The information shared by the instructor should be kept concise and brief. The skill of ‘teach general rules’ is not essential. It may be skipped when the learner has provided all relevant information [2].

This microskill should be used to teach the logical and methodical approach to clinical interactions and may need the experience to be broken down into components that will aid the understanding required by veterinary learners [1]. It should be noted that in a veterinary study addressing this issue, it was disappointing to find that instructors were not engaged in explaining how to break down a problem [1].

- *‘With any case of abdominal distention, it is good to think of the ‘6 Fs of abdominal distention’. The instructor should ask the learners to list them: fat (any tissue), flatus (any gas), fluid, fetus, food (ingesta or feces), and foreign body.*
- *‘In the selection of an appropriate medication, it is common to look for information. However, rather than looking in textbooks, I have found it more useful to use a veterinary drug handbook or internet resource. The textbook is always at risk of being outdated.’*

A common problem with the novice instructor is trying to show off and teach everything in a single case. This usually overwhelms the learner, and the integration of knowledge is interrupted. To prevent this problem from recurring, it may be useful for the instructor to think that ‘if it is impossible to cover it in about one minute, it is probably too much’.

### 2.4. Feedback Microskills—Reinforce What Was Done Right

This microskill should be used whenever a learner has handled the situation in a manner beneficial for the patient, client, colleagues, or the clinic, in particular if the behavior can be repeated [36]. With this microskill, the instructor uses the technique of positive feedback.

- *‘It is evident you have considered the cost-benefit of the applicable tests to assist with the definitive diagnosis of this population level problem. Your approach has certainly impacted the decision to go ahead with submitting the samples with the confidence they will return information to be used in the management of the current problem and prevention of recurrence in subsequent years.’*
- *’Consideration of the age and hydration status of the patient in the decision on the dosage and frequency of administration certainly decreased the risks of liver and kidney failure of this patient.’*
- *‘Specifically, you did an excellent job in considering the age of this calf, being factually non-ruminant. This prevented an unnecessary discussion on how appetite in ruminants results in alterations in the rumen microbiota.’*

### 2.5. Feedback Microskills—Correct Mistakes

This microskill should be limited to only a few sentences to correct erroneous behaviors (and fill in gaps in knowledge) by the learner that were detected during the clinical interaction as part of the approach to arrive to a diagnosis or a management plan. In this microskill, instructors use the technique of corrective feedback. Whenever possible and as required, the discussion considering the mistake in behavior should include what was done wrong and how that action has impacted others. Adhering to the principles of a ‘safe learning environment’ and ‘provide effective feedback’, the approach to the discussion on correcting the erroneous behavior should be what was done ‘not the best’ rather than ‘badly’.

It is human nature to react impulsively and correct mistakes as they occur. Therefore, whenever possible, instructors should resist the urge to correct the mistake as it occurs, with this microskill used last. It is possible that the learner would already have detected the mistake that needed corrective behavior. Self-assessment and self-reflection are indeed important skills for a learner. Even if the mistake is not acknowledged, the correct approach would be to ask the learner to critique his/her actions. This may prompt discovery that the learner has already detected the mistake. When a learner acknowledges the mistake and proposes solution(s) preventing it from recurring, there is no need to ’correct’ the mistake. It is also possible that a learner acknowledges the mistake but cannot find a solution(s). Hence, the learner may seek recommendations from peers and/or instructors on how to prevent the behavior recurring. Additionally, for some mistakes in behavior, as a part of the ‘safe learning environment’ this microskill may need to be carried out at an appropriate time and place, possibly waiting until a calmer, more private setting becomes available.

## 3. Limitations of the Five-Microskills Model

The FMS model has some limitations. First, it is an instructor-based rather than a learner-based model of clinical teaching. Second, the FMS model lacks the debrief of the clinical teaching interaction. Third, it lacks the provision of addressing the physiological/psychological state of the learner [36]. Fourth, the model does not provide solutions for poorly collected data and requires the instructor to be present. Fifth, although used in human emergency medicine settings [53,54,55], it may not be suited for all situations (e.g., life-threatening emergencies).

## 4. Debrief

The debrief on clinical teaching interactions is assumed to be an essential part of clinical teaching. Whilst it is not directly a part of the five-microskills model, it is an important aspect for learning. The debrief helps guide a learner on how to differentiate between important and non-important findings, e.g., the relevance of a fever in a patient with signs of gastrointestinal tract (GIT) involvement. It helps the learner discover reasons why certain information or data collection approaches may only be useful at certain times and not others, and provides an opportunity to discuss alternative approaches without devaluing what the learner has proposed or carried out. It also provides an opportunity for the learner to reflect on the clinical interaction and consider the learnings from that interaction.

- *‘Can you summarize what you learned at this farm visit, considering hoof health/reproductive performance/udder health?’*

## 5. Further Research Required

Randomized clinical studies testing the efficacy of the FMS model in veterinary clinical education are required. Of particular interest should be the level of clinical reasoning of learners exposed to trained or non-trained instructors, the effect of omitting one or more of the microskills, the effect of the team structure (e.g., experience of instructor, learners of different knowledge levels), and the time spent on teaching compared with ‘routine clinical intervention’. Similar strategies apply to studies comparing the efficacy of other clinical teaching models (e.g., the SNAPPS model).

## 6. Clinical Interaction Example

### 6.1. Description of the Clinical Interaction

The setting of the clinical interaction: a farm call out. Case of chronic abdominal distention in a 7-year-old Jersey cow.

Learning objectives during this clinical interaction should be having the learner demonstrate professionalism (The Royal College of Veterinary Surgeons; RCVS day one skills 3 and 4), self-awareness and self-reflection (RCVS day one skills 9, 13 and 14), effective communication (RCVS day one skill 17), clinical reasoning (RCVS day one skills 22 and 24), and full clinical examination (RCVS day one skill 29), whilst showing understanding of the ethical, legal, and prescribing responsibilities (RCVS day one skills 2 and 7) and promoting the health and safety of the patient and applying risk management to practice (RCVS day one skill 16) [56]. Veterinary learners are aware that clinical reasoning is not synonymous with pattern recognition and that the need of a logical and methodical approach to a clinical interaction is required [1].

The learner carries out a relatively good health interview and a good clinical examination. Additionally, the learner summarizes the findings succinctly. Important health interview information includes a prior surgery for a traumatic reticulo-peritonitis (two years ago) and that the cow has been unwell for about 3 weeks and is suspected to be pregnant. Important clinical findings include atonic rumen, lack of abdominal tenderness, last trimester pregnancy, and a slight bradycardia. The learner describes the distention as being obvious in both left quadrants and the ventral right quadrant of the abdomen (instructor’s reasoning: ‘L’- or papple-shaped). When asked for a diagnosis, the learner responds that it is a case of an ascites (instructor’s pattern recognition: ‘vagus indigestion’; instructor’s reasoning: resulting from a trauma to the vagus nerve, based on the previous surgical intervention).

### 6.2. Get a Commitment

- *‘Can you carry on as if I were not here.’*

This passes the responsibility of data collection completely to the learner.

### 6.3. Probe for Supporting Evidence

- *‘Can you explain what you mean by a slight bradycardia and tell me how you would explain this finding.’*

### 6.4. Teach General Rules

- *‘As a rule of thumb, all mature cattle with abdominal pain will have tachycardia and/or arrhythmia.’*

### 6.5. Feedback—What Was Done Right

- *‘Well done with the health interview and the clinical examination. Your succinct description allowed you to create a relatively narrow differential diagnosis list.’*

### 6.6. Feedback—Correct Mistakes

- *‘I completely agree that this is a case of an abdominal distention. However, your tentative diagnosis of ascites may need re-thinking. You described the location of the distention to be in both left quadrants and the ventral right quadrant. Missing the main pathophysiologic cause, at a minimum, would result in an inappropriate management recommendation and, perhaps, a disgruntled client calling back for a re-visit. As a worst case, the cause of the abdominal distention may compromise the function of the vital organs and the patient may die. So, before making a rushed decision on the potential diagnosis with any case of abdominal distention, consider all pathologic mechanisms. Now, when we have discussed this, can you think of what causes of chronic abdominal distention should be considered in this cow?*

*The ‘…can you think of what causes of a chronic abdominal distention should be considered in this cow?*’ is actually, again, the use of the ‘get a commitment’ microskill.

## 7. Teaching Practical Sessions Example

### 7.1. Description of the Practical Session

Urine assessment in a clinical biochemistry practical. Summary of the outcomes is provided in the table below (Table 2). 

Undeniably, in a real-life situation, other combinations of outcomes may be obtained. However, covering all possible scenarios is beyond the scope of this article.

Learning objectives for this practical session should be that the learner demonstrates the ability to carry out an appropriate diagnostic test, to interpret and understand the limitations of the test results (RCVS day one skill 31), and to promote the health and safety of people and environment (RCVS day one skill 44).

### 7.2. Get a Commitment (Learners 1–5)

- *‘Can you apply all tests you have learned and are applicable to a urine sample and write your results for each test on the answer sheet on front of you? It would be good if you could also write the interpretation of the findings.’*

### 7.3. Probe for Supporting Evidence (Learners 1 and 2)

- *‘I can see you have arrived at the result of hemoglobinuria in this urine sample. Can you briefly explain how you got there?’*

**Learner 1 only:**

- *‘Can you think of tests that would confirm your finding?’*

The *‘Can you think of tests…’* is a second use of the ‘get a commitment’ microskill. It is obviously beyond the level of comfort of early year learners, but it will stimulate self-directed learning.

### 7.4. Teach General Rules (Learners 1–5)

- *‘Red urine’ is a common finding in animal species and may be caused by the presence of any of several pigments. Some of these are physiologic, such as feeding beetroot to cattle. However, the presence of some pigments is indicative of an abnormal condition occurring in the body. For example, in cattle, causes of ‘red urine’ may include hematuria, hemoglobinuria, myoglobinuria, and porphyrinuria.’*

### 7.5. Feedback (Learners 1–5)

#### 7.5.1. What Was Done Right (Learner 1)

- *‘Well done in diagnosing hematuria using the combination of the urine dipstick and microscopic examination of the sediment. Causes of ‘red urine’ are many and each has a specific management approach. The correct diagnosis of the pathophysiologic mechanism of ‘red urine’ will allow you to narrow the list of your differential diagnosis. It would be good to think of the potential causes or the differential diagnosis list for hematuria.’*

The *‘It would be good to think of the potential causes or the differential diagnosis list for hematuria.’* is again use of the ‘get a commitment’ microskill. It is obviously beyond the level of comfort of early year learners but will stimulate self-directed learning.

#### 7.5.2. Correct Mistakes (Learners 2–5)

**Learner 2:**

- *‘You did well in conducting the tests and have properly recorded the results. Unfortunately, it seems that you could improve in the interpretation of the findings. Yes, you are correct when you say that you detected a positive reaction on the blood indicator on the urine dipstick. You also mentioned that you found no cells and a lot of casts on microscopy. The test results were interpreted correctly. However, I am afraid the cause for the ‘red urine’ was interpreted incorrectly. Causes of ‘red urine’ are many and each has a specific management approach. An incorrect diagnosis of the cause of ‘red urine’ may result in an inappropriate management plan. Can you think of what else may cause a positive reaction on the blood indicator on the urine dipstick but has no red blood cells on microscopic examination of the sediment?’*

The *‘Can you think of what else may cause a positive reaction...’* is once again the use of the ‘get a commitment’ microskill.

**Learner 3:**

- *‘Yes, you are correct when you say that you detected a positive reaction on the blood indicator on the urine dipstick and found no cells and a lot of casts on microscopy, but you did not connect these at all. Despite correct test findings, you suspected a case of dietary pigmenturia. Dietary pigmenturia would not yield a positive reaction on the urine dipstick, and no cells or casts on the microscopic examination of the sediment are expected, as the dietary pigment should cause no damage to the kidney. Causes of ‘red urine’ are many and each has a specific management approach. An incorrect diagnosis of the cause of ‘red urine’ may result in an inappropriate management plan. Can you think of what may cause a positive reaction on the blood indicator on the urine dipstick, no presence of red blood cells, and ample amount of casts on microscopic examination of the sediment?’*

The *‘Can you think of what may cause a positive reaction...’* is again the use of the ‘get a commitment’ microskill.

**Learner 4, who does not recognize the problem:**

- *‘It seems that the tests were completed correctly. However, most likely, results were not recorded correctly. Hence, the interpretation of the tests may not be valid. An incorrect diagnosis of the pathophysiologic mechanism resulting in the ‘red urine’ would result in an inability to prepare an appropriate differential diagnosis list and, consequently, inappropriate management of the case. We can arrange to repeat the tests in your own time. Would you like to do that?’*

The *‘Would you like to do that?’* is again the use of the ‘get a commitment’ microskill.

**Learner 4, who does recognize the problem:**

- *‘As you mentioned, you suspect that you did not record the test results correctly. You also proposed to repeat the tests in your own time and seek peer-discussion time to assist you. If there are still problems, you would seek assistance from an instructor. Am I right?’*

**Learner 5:**

- *‘Obviously, something has gone wrong during your testing. This has resulted in incorrect results and an inability to interpret the problem correctly. An incorrect diagnosis of the pathophysiologic mechanism resulting in the ‘red urine’ would result in an inability to prepare an appropriate differential diagnosis list and, consequently, inappropriate management of the case. So, we have two options. One is to repeat the testing on your own in your own time. The other is to repeat the testing assisted by some of your peers. What would you like to do?’*

The *‘What would you like to do?’* is again the use of the ‘get a commitment’ microskill.

## 8. Conclusions

Veterinary instructors can use the FMS model of clinical teaching in a variety of teaching activities, both in traditional academic and clinical settings. The model is easy to understand and implement, and it has been proven effective in developing clinical reasoning skills of learners in human medical education. We see no reason the model cannot be integrated into veterinary medical education.

## Figures and Tables

**Table 1:** Application of learning preferences to the five-microskills model (adapted from Delzell [14]).

*Microskills Step*	Strategy	Learner Type
Get a commitment (‘What do you think?’)	Suggest learners write ideas down before the precepting encounter	Visual
	Allow time to formulate the response	Auditory
	Allow options for physical movement	Tactile
Probe for supporting evidence (‘Why do you think this is the case?’)	Suggest algorithms to provide mapping options	Visual
	Have learner use a whiteboard	Visual/Tactile
	Feed responses back to the learner using reflective listening	Auditory
Teach general rules (‘When this happens, do x’)	Use charts/graphs/tables Use mnemonics	Visual Visual or auditory (depends on type)
	Have learners read references/guidelines aloud	Auditory
	Suggest learners use color-coded markers on a whiteboard	Tactile
Reinforce what was right (‘Specifically, you did x well’)	Have learners write down key points in a notebook for future reference	Visual/Tactile
	Breakdown the process into component parts	Tactile
	Suggest learners feedback to their peers	Auditory
Correct mistakes (‘Next time this happens, do x’)	Elicit questions/ideas	Auditory
	‘Map’ it using a chart	Visual
	Have learners find a reference, or guideline	Tactile

**Table 2:** Urine assessment results of learners and their interpretation.

Learner	Test Carried Out	Test Result	Result Interpretation	Explanation of Interpretation
1	Correct	Correct	Correct	Able to interpret
2	Correct	Correct	Correct	Unable to interpret
3	Correct	Correct	Incorrect	NA
4	Correct	Incorrect	NA	NA
5	Incorrect	NA	NA	NA

## Data Availability

Not applicable.

## Glossary

***Clinical encounter***	*any physical or virtual contact with a veterinary patient and client (e.g., owner, employee of an enterprise) with a primary responsibility to carry out clinical assessment or activity.*
***Clinical instructor***	*in addition to the regular veterinary practitioner’s duties, a clinical instructor should fulfil roles of: assessor, facilitator, mentor, preceptor, role-model, supervisor, and teacher of veterinary learners in a clinical teaching environment. Apprentice/intern in the upper years, Resident, Veterinary educator/teacher, Veterinary practitioner.*
***Clinical reasoning***	*process during which a learner collects information, process it, comes to an understanding of the problem presented during a clinical encounter, and prepares a management plan, followed by evaluation of the outcome and self-reflection. Common synonyms: clinical acumen, clinical critical thinking, clinical decision-making, clinical judgment, clinical problem-solving, and clinical rationale.*
***Clinical teaching***	*form of an interpersonal communication between a clinical instructor and a learner that involves a physical or virtual clinical encounter.*
***Deep learning***	*aiming for mastery of essential academic content; thinking critically and solving complex problems; working collaboratively and communicating effectively; having an academic mindset; and being empowered through self-directed learning.*
***Proper learning or a safe learning environment***	*an environment in which a learner feels safe, relaxed, and willing to take risks in pursuing a goal; enhances self-esteem and encourages exploration.*
***Self-directed learning***	*learners take charge of their own learning process by identifying learning needs, goals, and strategies and evaluating learning performances and outcomes.*
